# pH-responsive and targeted delivery of curcumin via phenylboronic acid-functionalized ZnO nanoparticles for breast cancer therapy

**DOI:** 10.1016/j.jare.2019.02.036

**Published:** 2019-03-01

**Authors:** Mousumi Kundu, Pritam Sadhukhan, Noyel Ghosh, Sharmistha Chatterjee, Prasenjit Manna, Joydeep Das, Parames C. Sil

**Affiliations:** a Division of Molecular Medicine, Bose Institute, P-1/12, CIT Scheme VII M, Kolkata 700054, India; b Biological Science and Technology Division, CSIR-North East Institute of Science and Technology, Jorhat, Assam 785006, India; c School of Chemistry, Shoolini University of Biotechnology and Management Sciences, Bajhol, PO Sultanpur, Distt. Solan 173229, HP, India

**Keywords:** Breast cancer, Curcumin, pH-responsive, Reactive oxygen species, Targeting, Zinc oxide nanoparticles

## Abstract

Nanoparticle-mediated targeted delivery of bioactive natural compounds has recently been gaining much interest for breast cancer therapy. Herein, phenyl boronic acid (PBA)-conjugated and pH-responsive ZnO nanoparticles (diameter ∼40 nm) were synthesized for the tumor tissue-specific delivery of curcumin. PBA conjugation facilitates the targeted delivery of curcumin to the sialic acid overexpressed in breast cancer cell membranes. Curcumin-loaded ZnO nanoparticles (ZnO-PBA-Curcumin) caused apoptotic cell death in MCF-7 human breast cancer cells by inducing oxidative stress and mitochondrial damage. Further, *in vivo* intravenous (i.v.) administration of ZnO-PBA-Curcumin was found to effectively decrease tumor growth in Ehrlich ascites carcinoma (EAC) tumor-bearing mice via the enhanced accumulation of curcumin. Interestingly, ZnO-PBA-Curcumin did not show any signs of systemic toxicity. The cytotoxic potential of the nanohybrid ZnO-PBA-Curcumin is attributed to the combinatorial cytotoxic effects of curcumin and ZnO in cancer cells. Collectively, ZnO-PBA-Curcumin may represent a potential treatment modality for breast cancer therapy. This study provides insight into the tumor cell targeting mechanism using PBA functionalization, and the anticancer efficacy of curcumin-loaded pH-sensitive nanohybrids can be attributed to the differential oxidative stress-inducing properties of curcumin and Zn^+2^ ions.

## Highlights

• A novel ZnO-PBA-Curcumin nanohybrid was synthesized.
• Targeted delivery was achieved in cancer cells through PBA functionalization.
• Loading curcumin onto nanoparticles increased its anticancer effects.
• The pH-dependent release of curcumin was obtained in cancer cells.
• ZnO-PBA-Curcumin nanohybrids exhibited significant anticancer activity without any systemic toxicity.

## Graphical abstract

**Figure:**
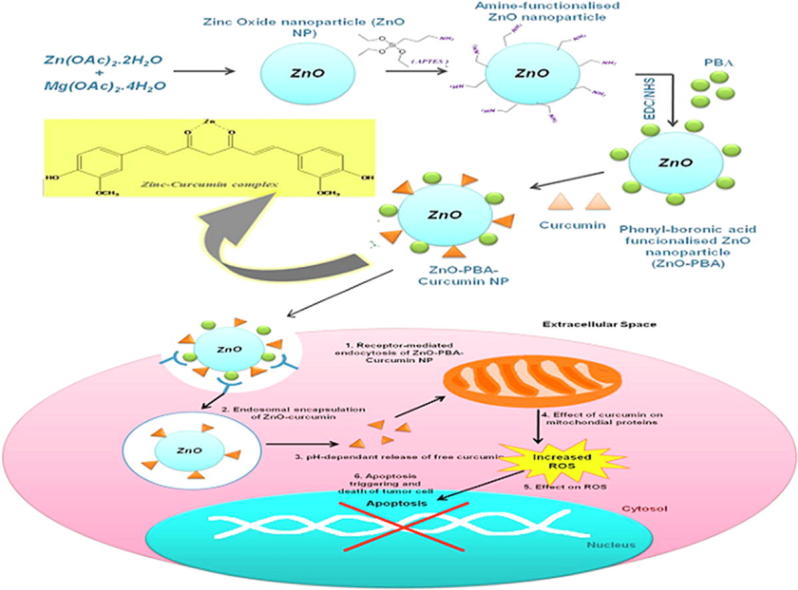

## Introduction

Cancer is a pathophysiological condition marked by uncontrolled cell division and the production of heterogeneous cell populations, thus forming a malignant neoplasm [1]. Several avenues have been explored for the treatment of this deadly disease, including chemotherapy, radiation, and surgery over the past several decades. Chemotherapy remains the first choice for breast cancer treatment, but deadly side effects resulting from these cytotoxic chemotherapeutics have posed hindrances along the way [2]. Nanoparticle-mediated drug delivery systems (DDS) have emerged as promising tools in this direction, as they can be utilized for the treatment of various diseases by circumventing healthy body tissues, thus causing minimal cytotoxicity and cell death in the healthy tissues while targeting only the diseased tissues [3]. Many nanoparticles themselves have anticancer properties, whereas others are described as nanocarriers used for ferrying the hydrophobic drugs selectively to the site of neoplasia [4]. Nanocarriers that have already been tested in this field include metal and nonmetal nanoparticles, magnetic nanoparticles, liposomes, solid lipids, dendrimers, polymers, niosomes and micelles. Nanocarriers can be targeted towards the cancer cells using specific ligands or antibodies, and they induce toxicity in cancer cells due to their inherent anticancer properties or the anticancer properties of the loaded drug or both, rendering them as multifunctional.

Metals and metal oxides are great nanoparticle choices because of their wide range of oxidation states, tunable size and shape, and surface chemistry. Zinc oxide nanoparticles (ZnO NPs) have been widely used in cosmetics as UV blockers [5] and in industrial tools as semiconductors and as photocatalysts. Recently, ZnO NPs have been reported to be a potent anti-cancer therapy in biomedical research owing to their attractive and distinguishable chemical and physical properties [6], [7], in addition to their applications in biosensing and imaging [8] and bacterial studies [9]. Previous studies with ZnO NPs reported that they could exhibit high cancer cell selectivity, retention [9], and controlled release of ligated as well as loaded drugs, besides being able to cross the therapeutic indices such as the other chemotherapeutic agents currently in use against cancer [10]. ZnO is biocompatible and easy to prepare from low-cost starting materials. It dissociates rapidly to Zn^2+^ at acidic pH values and revealing its cytotoxic properties [11], thereby showing its pH-responsive cytotoxicity. The tumor microenvironment is acidic, which is a well-recognized pathological feature of cancer. This attribute of cancer cells has been harvested for the controlled release of chemotherapeutics at the malignant site by acidic pH-responsive ZnO NPs, while sparing the healthy body tissues. Zn^2+^ results in mitochondrial dysfunction, ROS outburst and oxidative stress [7], lipid peroxidation and DNA damage [12], ultimately leading to cell death. However, the surface of ZnO NPs can also be passivated by the adsorption of small organic molecules (drugs) [13]. Coulombic, hydrophobic or covalent interactions might guide the entry of the drug-loaded ZnO NPs into the malignant cells by intracellular uptake mechanisms [14], and once it gains entry, the above event cascade occurs to ultimately kill the cancer cells.

Curcumin, a polyphenol obtained from the herb *Curcuma longa*, is a staple ingredient in the Asian culinary system. Research over the past couple of years has shown that it has various drug properties, including anti-inflammation [15], anti-cancer and anti-angiogenesis [16]. It has shown promising antitumor effects by inhibiting the proliferation of cancer cells and cytotoxicity by the induction of apoptosis and DNA damage. However, its poor solubility and absorption, low bioavailability [17], rapid metabolism and high elimination rate from systemic circulation hinders its use as a chemotherapeutic agent [18], similar to approximately 40% of the hydrophobic small molecule anti-cancer drugs currently marketed or in clinical trials [19]. Using nanomaterials is a promising avenue to address these shortcomings and to increase the efficacy of prospective drug candidates [20], [21]. As stated above, ZnO NPs are a very low cost but effective option, and when functionalized with a tumor-targeting molecule, such as phenyl boronic acid (PBA), folic acid, or hyaluronic acid, the nanodrug composite is expected to act with maximum efficacy on the cancer cells, thus causing cancer cell death [22].

In this study, for the first time, a PBA-conjugated nanohybrid was synthesized for the delivery of curcumin via targeting of the sialic acid residues overexpressed on the surface of breast cancer cells, and their anti-tumor efficacy was monitored using *in vitro* as well as *in vivo* tumor models.

## Material and methods

### Materials

Magnesium(II) acetate tetrahydrate (99%), zinc(II) acetate dihydrate (99.5%), N-hydroxyl succinimide (97%), 1-(3-dimethylaminopropyl)-3-ethyl carbodiimide hydrochloride (EDC∙HCl) extra pure (99%), 3-carboxybenzeneboronic acid (97%), potassium hydroxide pellets, disodium dihydrogen phosphate dihydrate, potassium dihydrogen phosphate, 3-(4,5-dimethylthiazol-2-yl)-2,5-diphenyl tetrazolium bromide (MTT) and sodium chloride were purchased from Sisco Research Laboratory (Mumbai, India). 3-aminopropyl triethoxysilane (APTES) with a purity of 99% was purchased from Sigma-Aldrich (St. Louis, Missouri, USA). Ethyl alcohol (EtOH) and dimethyl sulfoxide (DMSO) were purchased from Merck (Kenilworth, New Jersey, United States). Curcumin, DMEM media, RPMI-1640 media, amino acids and antibiotics were purchased from Hi-Media (Mumbai, India). Fetal bovine serum (FBS) was purchased from Thermo Scientific Hy-Clone (Logan, Utah, USA).

### Synthesis of amine-functionalized zinc oxide nanoparticles (ZnO NPs)

ZnO and ZnO-NH_2_ NPs were synthesized following previously reported procedures [11], [23]. First, a mixture of zinc acetate (2.0 mmol) and 44 mg of magnesium acetate were refluxed in 50 mL anhydrous ethanol for almost 5 h at 60 °C. To this mixture, an ethanolic solution of KOH (2.5 mmol) was added dropwise and the solution was stirred for 2 h at 60 °C. To obtain amine-functionalized nanoparticles, 1 mL of APTES) was added to the solution with stirring at room temperature for 1 h. The solid nanoparticles were obtained by centrifugation (6000 rpm for 20 mins) and washing several times with anhydrous ethanol.

### Tagging of 3-carboxybenzeneboronic acid (PBA) to ZnO NPs

After dissolving 100 mg of 3-carboxybenzeneboronic acid (PBA) in 10 mL DMSO, 40 mg of EDC and an equivalent amount of NHS were added for PBA activation. The reaction stirred for 3 h. Then, ZnO NPs were dispersed in DMSO and mixed with the activated PBA. After that, the solution was stirred for 24 h to obtain ZnO-PBA NPs.

### Loading of curcumin to ZnO-PBA NPs

The dispersed solution of ZnO-PBA NPs was added to a solution of curcumin in DMSO, and loading continued for 24 h with stirring. After loading, the nanosuspension was centrifuged and washed several times to separate the curcumin-bound nanoparticles. The supernatants were collected and pooled, and the amount of free curcumin present in the supernatant was determined from an absorbance measurement at 420 nm with a UV–visible absorbance spectrophotometer. A calibration curve was drawn using known concentrations of curcumin, and the drug loading content and entrapment efficiency were calculated as follows:

$$\text{Drug loading content}\left(\%\right)=\left[\left(\text{weight of drug in nanoparticles}\right)/\left(\text{weight of nanoparticles taken}\right)\right]\;\times \;100$$

$$\text{Drug entrapment efficiency}\left(\%\right)=\left[\left(\text{weight of drug in nanoparticles}\right)/\left(\text{weight of drug injected}\right)\right]\;\times \;100$$

### Characterization of synthesized nanoparticles

UV–visible spectra of ZnO-NH_2_ NPs were recorded by using a Shimadzu spectrophotometer. The hydrodynamic size and zeta potential of the prepared particles were measured by dynamic light scattering (DLS; Delsa™ Nano C particle size can analyzer, Beckman Coulter, Brea, CA, USA). The study of particle size and morphology of the whole nanohybrid were carried out using a transmission electron microscope (TEM; Zeiss-EM10C-100 KV, Japan) and a field emission scanning electron microscope (SEM; JSM7600F, JEOL, Japan). Fourier-transform infrared (FTIR) spectra of the synthesized nanoparticles were recorded in the region of 400–4000 cm^−1^ with an FTIR instrument (NEXUS-470, Nicolet, USA), using KBr pellets.

### pH-dependent release

A dialysis diffusion technique was used to determine the pH-sensitive release of curcumin from ZnO-PBA-Curcumin nanoparticles in different buffer solutions (pH 5, 6 or 7.4). In dialysis bags (MWCO 3500 Da), nanohybrids containing 1 mg/mL curcumin were suspended in 10 mL of 1× PBS with continuous shaking at 100 rpm and 37 °C. After a predetermined time interval of 0, 6, 12, 24, 36 or 48 h, 1 mL of the suspension was placed in a clean tube and replenished with fresh 1 × PBS in the dialysis membrane. Followed by this, the O.D. value of the suspension was determined using a UV–Vis spectrophotometer at 420 nm.

### Fluorescent micrographs of the nanoparticles

The newly synthesized ZnO NPs were characterized by fluorescence microscopy (fluorescence microscope, Nikon). ZnO NPs with concentration of 2 mg/mL were dispersed in a 1× PBS solution. Then, a drop of the solution was put on a clean glass slide. The image in a 40× objective lens was captured by the fluorescence microscope using the bright field, DAPI, FITC and RITC filters [24].

### Determination of intracellular uptake of nanoparticles

In a 35 mm cell culture dish, MCF-7 cells were plated at a density of 0.3 × 10^6^ cells/well on a sterile glass cover slip. At a confluency of 70%, the cells were treated with ZnO NPs or ZnO-PBA NPs for 3 h. After, the cells were washed with 1× PBS to remove free nanoparticles. Finally, using mounting media with DAPI, the cover slips were mounted on a glass slide. Using the FITC filter, images were captured by the fluorescence microscope [25]. Cellular uptake was also quantified by flow cytometry analysis [26].

### Cell culture

Human breast adenocarcinoma cells (MCF-7) and human breast epithelial cells (MCF-10a) were used in this study. The cells were obtained from the National Centre for Cell Science (NCCS, Pune, India) and cultured in Roswell Park Memorial Institute media (RPMI-1640) containing 10% fetal bovine serum (FBS) along with 1,00,000 U/L penicillin and 100 mg/L streptomycin in a humidified atmosphere of 5% CO_2_ in air at 37 °C.

### Cell viability assay

Against MCF-7 and MCF-10a cell lines, the cytotoxicity of ZnO NPs, curcumin and ZnO-PBA-Curcumin were estimated by the standard MTT assay [24]. Initially, the cells were seeded in 96-well culture plates at a density of 1 × 10^5^ cells/well. After 24 h of incubation (37 °C, 5% CO_2_), cells were treated with different concentrations of ZnO-PBA NPs (3.25–26 μg/mL), free curcumin (1.75–14 μg/mL) or ZnO-PBA-Curcumin (5–40 μg/mL) for 48 h. After removing the media, 100 µL/well of 0.5 mg/mL MTT was added. After 4 h incubation, 100 μL/well of dimethyl sulfoxide (DMSO) was added. By using a microtiter plate reader, the absorbance was measured at 570 nm.

### Measurement of intracellular reactive oxygen species (ROS)

To determine the intracellular ROS level, approximately, 2 × 10^5^ cells/well were plated and exposed to a specific dose of ZnO NPs, curcumin or ZnO-PBA-Curcumin. Then, cells were scraped and centrifuged (5 mins, 300*g* and room temperature). After suspension in 1 mL PBS, H_2_DCFDA (2,7′-dichlorodihydrofluorescein diacetate) was added to final concentration of 2 µM and incubated in the dark for 20 mins at 37 °C. Finally, by flow cytometry, the fluorescence emission was measured using a 525 nm bandpass filter [27].

### Measurement of mitochondrial membrane potential (MMP)

The determination of MMP was carried out following the previously described method [28]. JC-1 dye was used in this experiment. After incubation at 37 °C for 30 mins with 5 mM JC-1 dye, cells were centrifuged (5 mins, 300*g*) followed by suspension in PBS. Then, flow cytometric analysis of the fluorescently labeled cells was carried out using a BD FACSCalibur flow cytometry system (BD Biosciences).

### Determination of the mode of cell death

The *in vitro* mode of cell death was checked by FACS analysis [29]. Briefly, after exposure to ZnO-PBA NPs, curcumin, or ZnO-PBA-Curcumin, cells were suspended in annexin V binding buffer and stained with annexin V dye for approximately 30 min. After washing with 1× PBS, the cells were studied at an excitation wavelength of 485 nm and an emission wavelength of 530 using a BD FACS Calibur flow cytometry system.

### Development of the *in vivo* solid tumor model

In this study, 4–6 week old male Swiss albino mice (weighing ∼25 g) were used, and they were collected from the central animal house and research facility of the Bose Institute, Kolkata, India. Animals were acclimated for 14 days in alternating 12 h light/dark cycles and provided with water *ad libitum* and sterile food. *All animal experiments were carried out according to the guidelines of the Institutional Animal Ethics Committee (IAEC), Bose Institute, Kolkata [IAEC/BI/3(I) cert. /2010], and the experimental work plan concurred with the IAEC as well as the CPCSEA (Committee for the Purpose of Control and Supervision on Experiments on Animals), Ministry of Environment and Forests, New Delhi, India (1796/PO/Ere/S/14/CPCSEA).*

All animals were distributed into 5 sets (n = 6/set). To develop a solid tumor model (in sets II, III, IV and V), Ehrlich ascites carcinoma (EAC) cells were injected (10^7^ cells per 50 µL/mouse) into the left flank of the mice on day 0 [29]. EAC cells were not injected in set I. Ten days after injection, the animals were separated into sets II, III, IV and V, as follows:

Set I- Control
Set II- Untreated tumor (EAC)
Set III- Treated-1 (EAC + ZnO NPs treatment at a dose of 18.6 mg/kg body weight)
Set IV- Treated-2 (EAC + ZnO-PBA-Curcumin treatment at a dose of 28.6 mg/kg body weight)
Set V- Treated-3 (EAC + curcumin treatment at a dose of 10 mg/kg body weight)

Then, the animals were treated intravenously with ZnO, ZnO-PBA-Curcumin or curcumin on alternate days for 14 days (Table 1). After that, the animals were sacrificed, the tumors were removed, and liver, kidney, and spleen tissues were collected. The tumor volume was calculated by using the ellipsoid volume equation. Tumor mass were determined using an electronic weighing balance. By comparing the morphologies of the spleen samples, the tumor-associated splenomegaly was detected in the experimental animals [30].

**Table 1:** Schematic representation of the nanoparticle administration protocol.

### Determination of curcumin accumulation in tissue

The quantitative accumulation of curcumin in tumor tissue was detected by HPLC. The tumor model was developed according to the protocol described above. Four tumor-bearing animals were taken and divided as set I: untreated tumor (animal received 0.9% NaCl solution); set II: ZnO-Curcumin; set III: ZnO-PBA-Curcumin and set IV: free curcumin. The equivalent dose of curcumin injected was 10 mg/kg.

The accumulation of curcumin was measured in the tumor tissue 24 h after administration of drug. The mice were sacrificed after the experimental time period, and the tumors were removed. A total of 100 mg of tumor tissue was taken from each animal and homogenization was performed in 0.05% DMSO and 75% EtOH. Then, the mixture was centrifuged for 15 mins at 10,000 rpm and the supernatant was dried in a nitrogen evaporator. The dry product was dissolved in MeOH and was loaded onto a C_18_ column. The amount of curcumin present was determined by reverse-phase HPLC using an isocratic system with a flow rate of 1.7 mL/min, a column temperature of 33 °C, a mobile phase of acetonitrile and 2% acetic acid (40:60, v/v), a detection wavelength of 428 nm and an injection volume of 20 µL [31]. Standard solutions of curcumin were prepared with concentrations of 5, 20, 30, 60, 80 and 100 µg/mL in 50% acetonitrile. The calibration curve of curcumin was linear within the above concentration range (regression equation: *y* = 34265*x* + 282273, R^2^ = 0.998). The limit of detection (LOD) and limit of quantification (LOQ) were 6.18 and 18.54 µg/mL, respectively. The recovery percentages for the 5, 20, 30, 60, 80 and 100 µg/mL concentrations were 99.68%, 98.67%, 104.23%, 93.36%, 106.79% and 89.4%, respectively.

### Immunohistochemistry

Immunostaining was performed on tumor tissue sections from experimental animals to check the expression of cleaved caspase-3 and caspase-9. The tumor tissues were removed from the mice, then processed and embedded into paraffin blocks following standard protocol [32]. The tissue blocks were cut into thin sections and adhered onto poly-L-Lysine coated glass slides. The sections were then deparaffinized, rehydrated and incubated with caspase-3 and caspase-9 antibodies (Cell Signaling Technology Inc., Danvers, MA). Horseradish Peroxidase (HRP)-tagged secondary antibodies (Abcam, Cambridge, UK) were added and incubated. The HRP substrate 3,3′-diaminobenzidine tetrahydrochloride (DAB) was then added, and the sections were counterstained with hematoxylin, dehydrated, and mounted in distyrene, a plasticizer, and xylene (DPX). Finally, the sections were observed under a microscope using a 20× objective lens.

### Histology

The flank tissues (tumors) from the control and experimental mice were fixed in 10% buffered formalin and processed for paraffin sectioning. Paraffin-embedded sections of approximately 5 µM thickness were stained with hematoxylin and eosin to study the histological changes under a light microscope [34].

### Analysis of systemic toxicity

For the detection of the systemic toxicity which may arise from the treatment with nanoparticles, animals were grouped in 8 sets (n = 3/set). To develop a solid tumor model (in sets II, III, IV and V), EAC cells were injected (10^7^ cells per 50 µLmouse) into the left flank of the mice on day 0. Sets I, VI, VII, and VIII comprised nontumor bearing mice, as follows:

Set I- Control
Set II- Untreated tumor
Set III-ZnO treated tumor
Set IV- Curcumin treated tumor
Set V- ZnO-PBA-Curcumin treated tumor
Set VI-ZnO treated
Set VII-Curcumin treated
Set VIII- ZnO-PBA-Curcumin treated

Then, the animals were treated intravenously with ZnO, ZnO-PBA-Curcumin or curcumin on alternate days for 14 days. The equivalent dose of curcumin injected was 10 mg/kg (Table 1). After sacrificing, blood was taken from each mouse and the serum was isolated by centrifugation [33].

The levels of alkaline phosphatase (ALP) and alanine aminotransferase (ALT) were determined as hepatic toxicity markers, and blood urea nitrogen (BUN) and creatinine levels were determined as nephrotoxicity markers by following the protocols of the kits (Span Diagnostics Ltd., India) [35]. For the estimation of systemic toxicity, liver and kidney tissues were removed from the experimental animals and histological examinations were carried out following the protocol mentioned above, using 40× magnification with a light microscope. In addition to this, the tumor-associated toxicity was observed by assessing the macroscopic morphology of the splenic tissue excised from the experimental animals.

### Statistical analysis

The results are shown as their mean ± SD, after performing at least three independent experiments. The statistical analyses were performed using one-way analysis of variance (ANOVA), and Tukey’s test was also performed to compare the mean values between the groups. The median and range were measured, and the Kruskal-Wallis test was performed for statistical analyses. A *P*-value of less than 0.05 was considered statistically significant.

## Results

### Synthesis and characterization of ZnO NPs, ZnO-PBA and ZnO-PBA-Curcumin

**Scheme 1:**
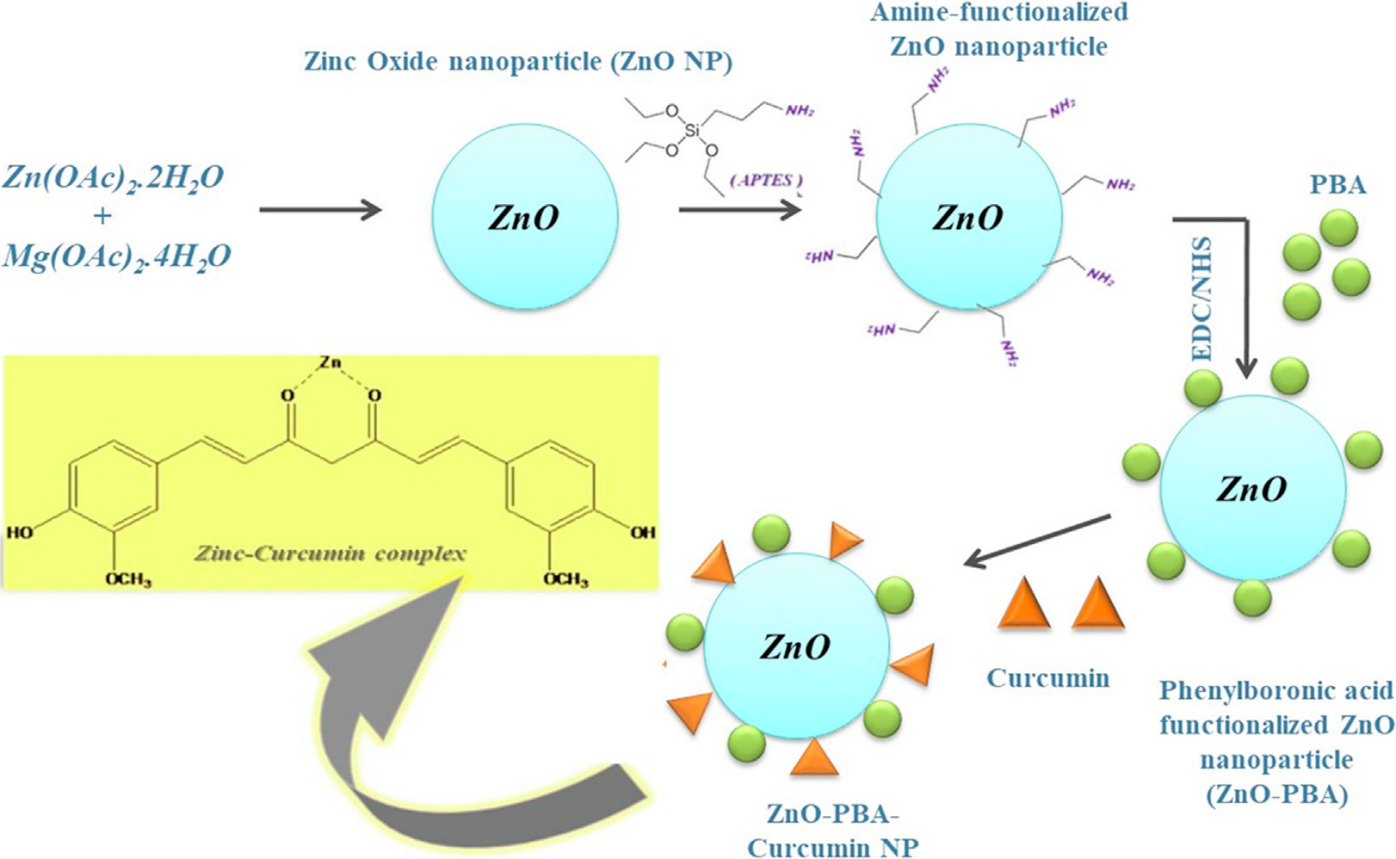
A schematic representation of the synthesis protocol for the nanohybrid.

First, ZnO NPs with a surface adsorption of amino groups were synthesized. To formulate ZnO-PBA, PBA was introduced onto the surface of ZnO, and lastly, curcumin was loaded (Scheme 1).

**Fig. 1:**
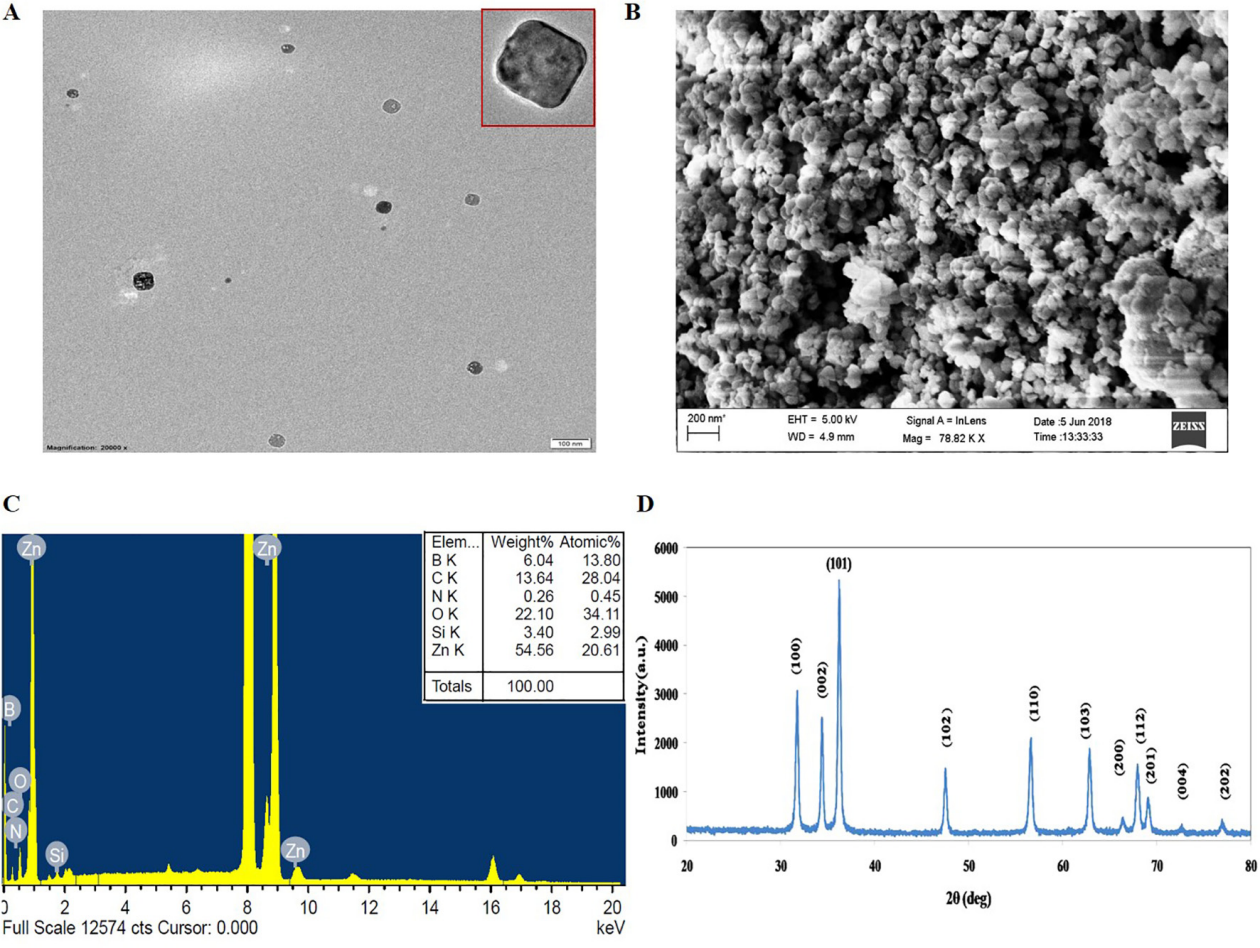
(A) TEM, (B) SEM, and (C) EDX of ZnO-PBA-Curcumin Nanohybrids. (D) XRD analysis of ZnO NPs.

The DLC and DEE were found to be 35% and 27%, respectively (Table 2). To determine the size and shape of the ZnO-PBA-Curcumin nanohybrids, TEM and SEM analyses were performed and are depicted in Fig. 1A and B.

**Table 2:** Drug loading content (DLC) and drug entrapment efficiency (DEE) of ZnO-Curcumin nanoparticles.

Feed Ratio, ZnO: Curcumin	DLC%	DEE%
1:2	35	27

TEM analysis was also performed for ZnO NPs (Fig. S1). A tetragonal morphology of the particles was observed with an average size of 30–40 nm. There was no significant difference in size among ZnO and ZnO-PBA-Curcumin as observed from the TEM study. The EDX spectrum of the nanohybrid is given in Fig. 1C. From this result, it is clear that the nanohybrid carried approximately 54.56% Zn, 22.10% O and 13.64% C by weight; silicon, boron and nitrogen were also present, indicating a successful surface functionalization of ZnO NPs. In Fig. 1D, the XRD pattern of the synthesized ZnO NPs is shown. All obtained peaks indicate the hexagonal crystalline structure of ZnO NPs, devoid of any impurities. XRD analysis was also done for ZnO-PBA and ZnO-PBA-Curcumin (Fig. S2), which showed hexagonal crystalline structures as well.

**Fig. 2:**
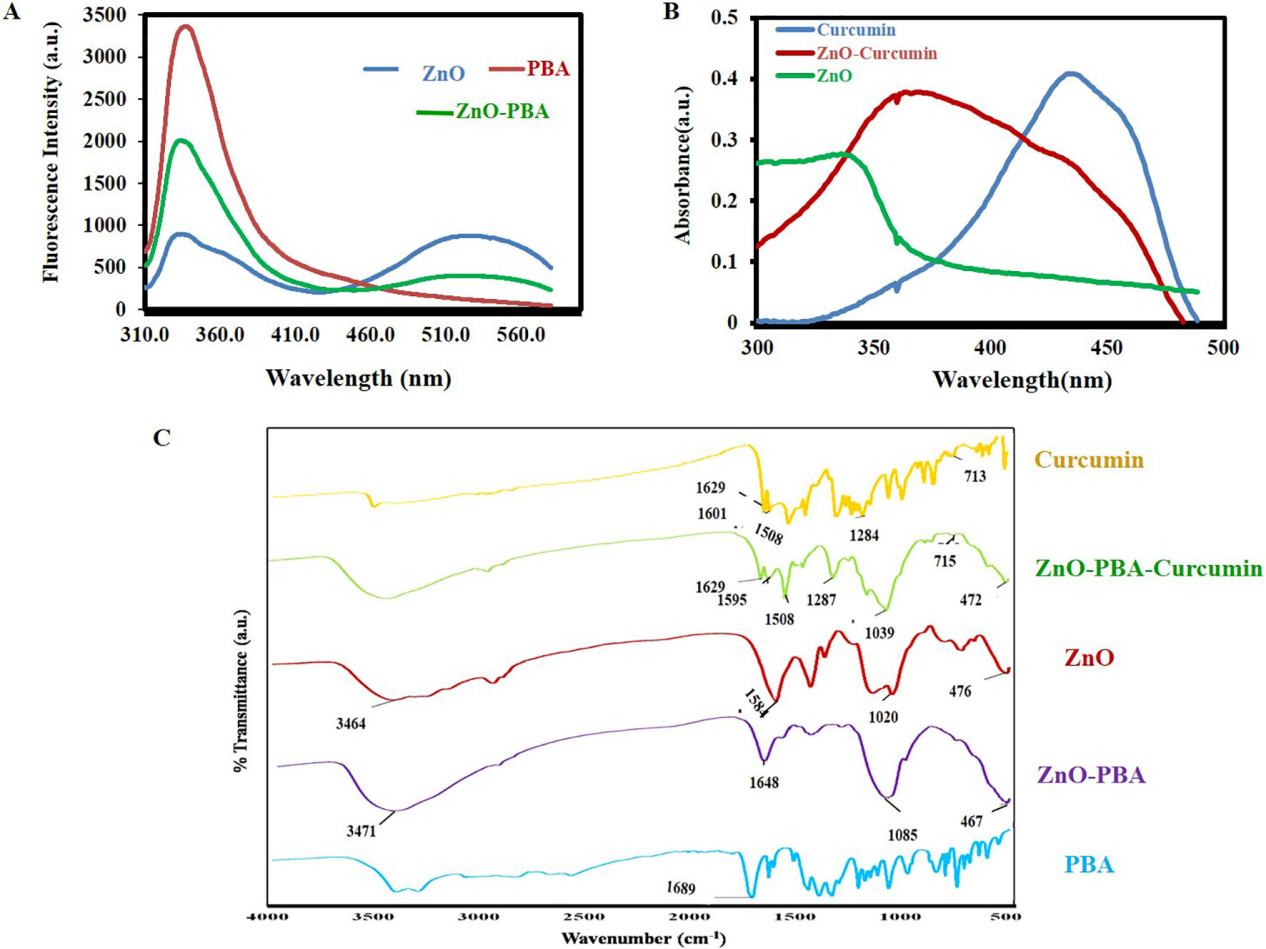
(A) Fluorescence spectra of ZnO, PBA and ZnO-PBA NPs. (B) UV–visible spectra of curcumin, ZnO and ZnO-Curcumin NPs. (C) FTIR spectra of curcumin, ZnO-PBA-Curcumin, ZnO, ZnO-PBA and PBA.

In Fig. 2A, the fluorescence spectra of ZnO NPs, PBA and ZnO-PBA are presented. Two strong emission peaks were obtained at 340 nm and 527 nm for both ZnO and ZnO-PBA after excitation at 310 nm. However, in the case of PBA alone, one strong emission peak at 340 nm was observed at the same excitation wavelength. The UV–visible absorption spectra of curcumin, ZnO NPs and curcumin-loaded ZnO NPs are presented in Fig. 2B. ZnO NPs showed a strong absorption peak at 341 nm. Pure curcumin showed a characteristic peak at approximately 430 nm, and a broad band was observed in case of curcumin-loaded ZnO NPs, probably because of the overlap of the two absorption peaks for pure curcumin and ZnO NPs.

In Fig. 2C, the FTIR spectrum of the nanoparticles is displayed. ZnO nanoparticles showed a peak transmittance at 476 cm^−1^. ZnO also showed transmittance at 1584 cm^−1^ and 1020 cm^−1^. A transmission peak at 1689 cm^−1^ was observed which is a characteristic peak of PBA. However, when PBA reacted with the —NH_2_ group of ZnO, the peak at 1689 cm^−1^ was missing, and a new peak was observed at 1648 cm^−1^. The transmission peaks of pure curcumin were observed at 1629 cm^−1^, 1601 cm^−1^, 1284 cm^−1^ and 713 cm^−1^, which could be attributed to the stretching vibration of predominantly mixed —C=C and —C=O bonds, symmetric aromatic ring stretching vibrations, enol C—O stretching vibrations and the cis C—H bond vibrations of an aromatic ring, respectively [26]. In curcumin-loaded ZnO NPs, the transmission peaks observed were similar to those of pure curcumin.

**Fig. 3:**
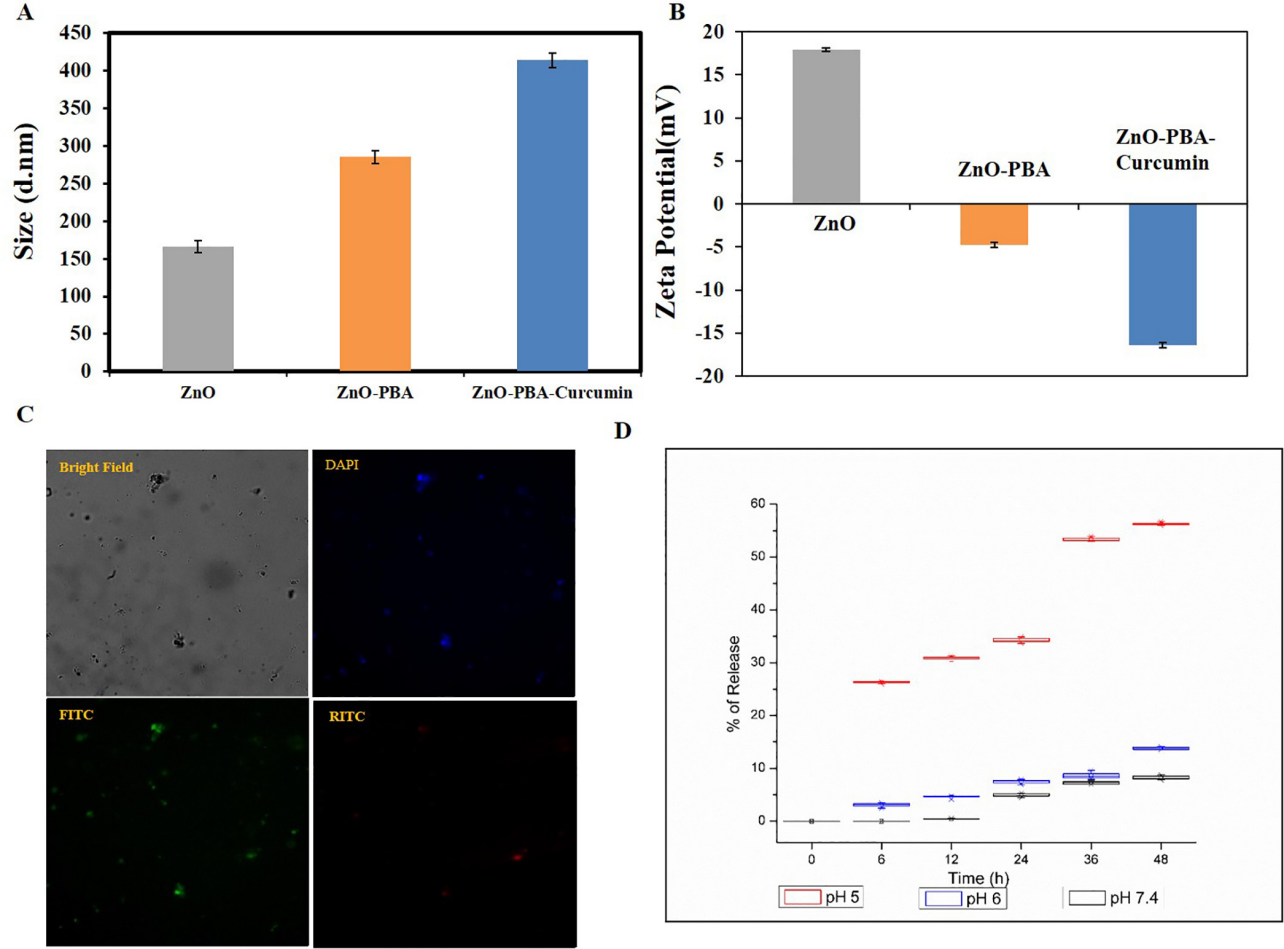
(A) DLS and (B) zeta potential of ZnO, ZnO-PBA and ZnO-PBA-Curcumin. (C) Fluorescence microscopy image of ZnO-PBA-Curcumin obtained at 40× magnification. (D) Time and pH-dependent release of curcumin from ZnO-PBA-Curcumin.

The average hydrodynamic diameters of ZnO, ZnO-PBA and ZnO-PBA-Curcumin were measured by dynamic light scattering (DLS). The hydrodynamic sizes of ZnO, ZnO-PBA, and ZnO-PBA-Curcumin were 166.3 ± 7.9 nm, 284.96 ± 8.3 nm and 413.63 ± 9.5 nm, respectively (Fig. 3A). In Fig. 3B, the zeta potentials are shown. The zeta potential values of ZnO NPs, ZnO-PBA and ZnO-PBA-Curcumin were 17.9 ± 0.2 mV, −4.7 ± 0.31 mV and −16.4 ± 0.30 mV, respectively.

The synthesized nanohybrids showed strong green (FITC) and blue (DAPI) fluorescence under a fluorescence microscope (at a magnification of 40×), which indicates their fluorescent nature (Fig. 3C). As a result, this nanohybrid can also be used as a potential bio-imaging agent.

### Time-based pH-dependent curcumin release study

Curcumin release from the nanohybrid was investigated in PBS at three different pH values (5.0, 6.0, and 7.4) and is depicted in Fig. 3D. Curcumin release was detected using UV–visible spectroscopy. The release of curcumin from the nanohybrid improved with decreasing pH. At pH 5.0, almost 56% of the curcumin was released after 48 h. The release percentages of curcumin were 13.8% at pH 6.0 and 8.2% at pH 7.4. After 48 h, no significant increase in the release percent was observed; i.e., a plateau was reached (data not shown).

### Intracellular uptake efficiency of the PBA-conjugated nanoparticles

**Fig. 4:**
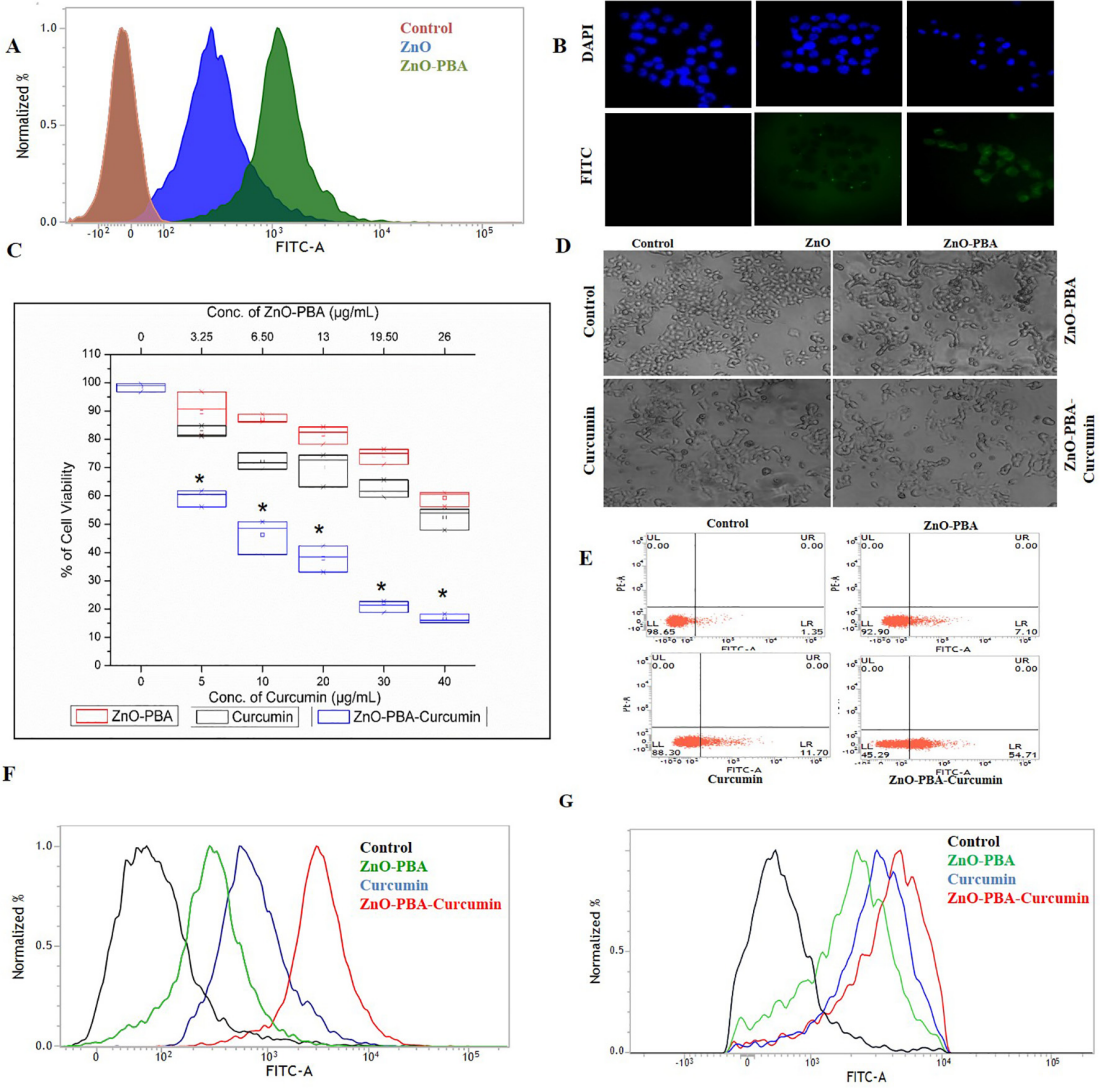
(A) and (B) Intracellular uptake of ZnO and ZnO-PBA NPs by FACS and fluorescence microscopy analysis in MCF-7 cells. (C) MTT cell viability. (D) Phase contrast micrographs obtained at 20× magnification. (E) FACS analysis with annexin V staining to determine the percent of apoptotic cells in the different experimental groups. The cell in the lower left quadrant denotes annexin V positive or apoptotic cells. (F) and (G) Determination of intracellular ROS levels and MMP by FACS analysis, respectively.

By using both fluorescence microscopy and FACS analysis, the intracellular uptake of the ZnO NPs and ZnO-PBA was determined in MCF-7 cells. After 3 h of incubation, the fluorescence intensity of the cells incubated with ZnO-PBA was found to be higher than that of ZnO NPs (Fig. 4A and B), indicating a higher cellular internalization for ZnO-PBA.

### Dose-dependent cytotoxicity of ZnO-PBA-Curcumin nanohybrids

The cytotoxic activity of the newly synthesized nanoparticles was measured using the MTT cell viability assay in MCF-7 cells. The dose-dependent cytotoxicity of ZnO-PBA-Curcumin was determined with a dose ranging from 5 to 40 μg/mL. ZnO-PBA-Curcumin exhibited a higher cytotoxic effect than equivalent concentrations of ZnO NPs and free curcumin (Fig. 4C).

The cytotoxic activity was also confirmed by studying the optical images of the nanohybrids and curcumin-treated cells. The incidence of cell shrinkage, membrane blebbing and the presence of apoptotic bodies gave a clear indication of the cytotoxicity of the nanohybrids. ZnO-PBA-Curcumin was again found to be more cytotoxic than both free curcumin and ZnO-PBA at equivalent concentrations (Fig. 4D). Further, FACS analysis with annexin V staining of MCF-7 cells showed that cells exposed to ZnO-PBA-Curcumin exhibited a higher percentage of apoptotic cells compared to cells exposed to equivalent concentrations of ZnO NPs or free curcumin (Fig. 4E). The nanoparticles and free curcumin were again found to be nontoxic to the normal MCF-10a cells (Fig. S3). No significant cytotoxicity was observed in MCF-10a cells for curcumin, ZnO-PBA and ZnO-PBA-Curcumin up to a concentration of 14 μg/mL, 26 μg/mL and 40 μg/mL, respectively, whereas profound cytotoxicity was observed in cancer cells at these concentrations.

### ZnO-PBA-Curcumin induced oxidative stress

FACS analysis was performed to measure intracellular the ROS levels and MMP in MCF-7 cells following ZnO-PBA-Curcumin exposure. To perform the intracellular ROS level and MMP studies, DCFDA and JC-1 dyes were used, respectively. In the study of intracellular ROS, the green fluorescence intensity in the ZnO-PBA-Curcumin treated cells was elevated compared to the control cells (Fig. 4F), indicating a higher level of ROS. This ROS level was also high in the case of ZnO-PBA-Curcumin treatment compared to ZnO-PBA or free curcumin treated cells at equivalent concentrations.

In the study with JC-1, the green fluorescence intensity in the ZnO-PBA-Curcumin-treated cells was enhanced compared to control cells (Fig. 4G), indicating MMP loss. The MMP loss was also high in the case of cells treated with ZnO-PBA-Curcumin compared to ZnO-PBA or free Curcumin treated cells at equivalent concentrations.

### Curcumin accumulation in tumor tissues

**Fig. 5:**
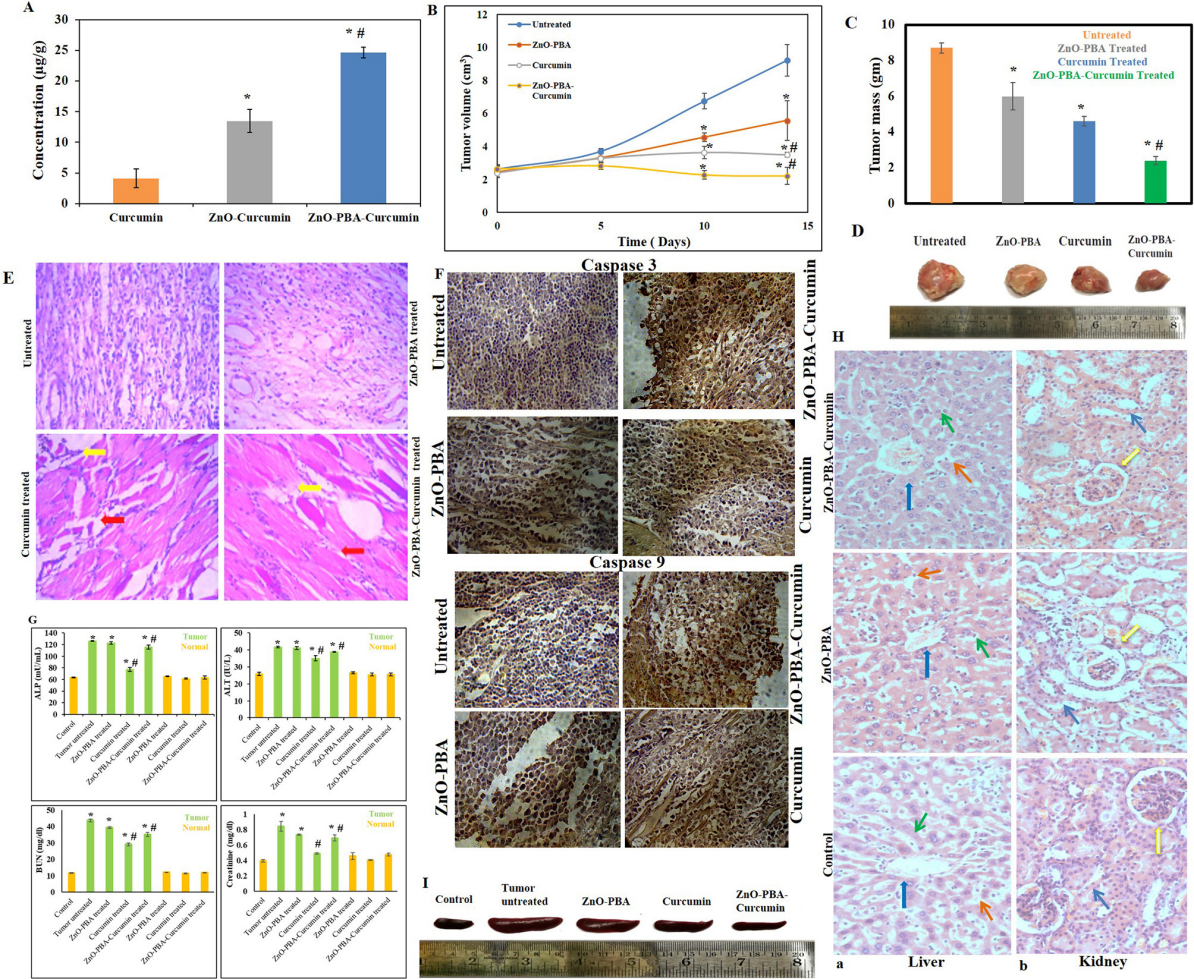
(A) Determination of tissue absorption of curcumin in tumor-bearing animals. “*” and “#” represent significant differences with respect to curcumin and ZnO-Curcumin treated groups, respectively, (P* < 0.05, *P*# < 0.05). (B) and (C) Tumor volume growth curve and tumor mass graph, respectively. All values are expressed as the mean ± SD, n = 6. “*” and “#” represent significant differences with respect to the untreated and curcumin-treated groups, respectively, (P* < 0.05, *P*# < 0.05). (D) Representative photographs of tumor tissues removed from different experimental groups. (E) Histological (H & E stained) overview of tumor tissue sections at 40× magnification. Yellow and red arrows indicate multinucleated tumor cells and tissue architecture, respectively. (F) Immunohistochemical detection of cleaved caspase-9 and cleaved caspase-3 in tumor sections. (G) Analysis of systemic toxicity (ALP, ALT, creatinine, and BUN levels) in different experimental animals. All values are expressed as the mean ± SD, n = 6. “*” and “#” represent significant differences with respect to the control and tumor control groups, respectively, (P* < 0.05, *P*# < 0.05). (H) Histological (H & E stained) overview of (a) liver (blue arrow: central vein; green arrow: hepatic cords consisting of hepatocytes; orange arrow: sinusoids) and (b) kidney (yellow arrow: glomerulus; blue arrow: renal tubules) tissue sections of control and treated mice at 40 × magnification. (I) Representative photographs of the spleen samples removed from different experimental groups.

The accumulation of curcumin in tumor tissue increased more with ZnO-Curcumin NPs treatment (13.4 µg/g) than with free curcumin treatment (4.1 µg/g). Again, increasing accumulation of curcumin in tumor tissue was obtained for the PBA-functionalized nanohybrid (24.6 µg/g) compared with the PBA nonfunctionalized ZnO-Curcumin (Fig. 5A).

### *In vivo* antitumor activity

The curcumin-loaded ZnO-PBA nanohybrid exhibited significantly enhanced antitumor activity in comparison to ZnO NPs and free curcumin in tumor-bearing mice. Intravenous injection of 10 mg/kg body weight of ZnO-PBA-Curcumin was found to reduce the tumor mass and volume more efficiently in comparison to the other treatment groups (Fig. 5B–D). We also calculated the tumor volume change among the several treatment groups over the experimental period of 14 days (Fig. 5B). We observed that in the untreated tumor control group, the tumor size increased continuously for 14 days. The ZnO-PBA treatment group showed a delayed increase in tumor volume over time. The curcumin and ZnO-PBA-Curcumin treatment groups showed no apparent change in tumor volume over time, although the best antitumor activity was obtained for the ZnO-PBA-Curcumin treatment group.

The tissue morphology of tumor-bearing mice showed complete disruption compared to control mice. In addition, the infiltration of multinucleated tumor cells was seen within the flank tissue of tumor-bearing mice, which was absent in the tissue of control mice. The tumor tissue morphology from the treated animals was moderately restored and they exhibited a significant reduction in infiltrating tumor cells in the following order: ZnO-PBA-Curcumin > curcumin > ZnO (Fig. 5E).

In addition to this, increased expression of both cleaved caspase-9 as well as cleaved caspase-3 was seen in the tissue sections of the ZnO-PBA, curcumin and ZnO-PBA-Curcumin administered animals compared to control tissue (untreated tumor-bearing animals) (Fig. 5F). The expression levels of both proapoptotic proteins was higher in the nanohybrid-treated group compared to the other experimental groups, indicating that the ZnO-PBA-Curcumin nanoparticles can significantly initiate the intrinsic apoptotic pathway leading to tumor cell death.

### *In vivo* systemic toxicity of nanoparticles

As described before, to assess nanoparticle-mediated hepatic and renal toxicities, the tissue histology and serum parameters were determined. It was found that solid tumor induction by EAC injection caused severe nephrotoxicity and hepatotoxicity. This is supported by the sharp increase in serum ALT and ALP activities in tumor-bearing mice (Fig. 5G). Additionally, increased BUN and creatinine levels also indicate poor renal health in these mice (Fig. 5G). However, treatment with the nanohybrid did not show any additional hepatotoxicity and nephrotoxicity over the nontumor and tumor control groups. Histological analysis also did not show any structural changes in the microenvironment of the liver (by analyzing the architecture of the central vein, hepatic cords and the morphology of the hepatocytes and sinusoids) and kidney tissues (by analyzing the structural organization of the glomerulus and renal tubules) of healthy animals after ZnO-PBA and ZnO-PBA-Curcumin treatment (Fig. 5H). Interestingly, in the case of ZnO-PBA-Curcumin treated animals, tumor-induced splenomegaly decreased compared to the untreated group (Fig. 5I).

## Discussion

In the present study, a novel ZnO-PBA-based drug delivery system was developed wherein a hydrophobic anticancer agent, curcumin, was loaded onto the surface of ZnO NPs (via metal ion-ligand coordination) for targeted drug delivery applications to cancer cells *in vitro* and to a tumor *in vivo*.

The prepared ZnO-PBA-Curcumin nanohybrids were characterized by TEM, SEM, EDX and FTIR spectroscopic analyses. We have compared the TEM images among ZnO, ZnO-PBA and ZnO-PBA-Curcumin NPs, but the apparent change in size was not significant. However, the apparent increase in size from ZnO to ZnO-PBA to ZnO-PBA-Curcumin was very clear from the DLS measurements. In TEM analysis, primary particle size is determined in a static and dried state, whereas in DLS measurements, the hydrodynamic size is determined from the diffusional properties of the dynamic nanoparticles in a solvated state. Therefore, the hydrodynamic diameter was larger than that obtained from TEM experiments because of the extensive solvation/hydration of the nanoparticles [36]. The adsorption of curcumin on the ZnO surface was confirmed by multiple characteristic peaks of curcumin obtained from the FTIR spectrum of curcumin-loaded ZnO NPs. The peak at 476 cm^−1^ represented the Zn–O bond stretching vibration. Transmittances at 1584 cm^−1^ and 1020 cm^−1^ for ZnO were designated as an N—H bending vibration and an Si—O—Si stretching vibration, respectively, pointing towards conjugation of APTES to ZnO nanoparticles. The peak for PBA at 1689 cm^−1^ appeared due to the —C=O bond vibration of the carboxylic acid group. In case of ZnO-PBA, the disappearance of this peak and appearance of a new peak at a lower energy gave evidence to the formation of an amide bond (—NH—CO—); i.e., PBA was immobilized onto the ZnO nanoparticle surface via covalent bond formation. The DEE and DLC contents were 27% and 35%, respectively. The successful surface modification of the ZnO NPs was also supported by the presence of silicon, boron and nitrogen in the EDX spectrum. A hexagonal crystalline structure of ZnO NPs with almost no impurities was confirmed by the obtained peaks [37].

The optical properties of the synthesized nanoparticles were examined by using both fluorescence and UV–visible spectroscopy. The coexistence of the emission peaks at 340 nm and 525 nm indicates the presence of both PBA and ZnO in the nanohybrid. The nanohybrid also showed a broad band covering the area of both pure curcumin and pure ZnO NPs due to their coexistence. However, the lower absorption values for curcumin in the nanohybrid were thought to be due to the presence of an interaction between curcumin and ZnO [38]. The process of PBA and curcumin attachment on the ZnO NPs surface was also monitored by DLS experiments and zeta potential analysis. The successive increase in the hydrodynamic size indicated the conjugation of PBA and curcumin in a stepwise manner. The reversal in the surface potential of the positively charged ZnO NPs indicated the attachment of negatively charged PBA molecules, and the further increase in negative charge is attributed to the presence of curcumin with several ionizable hydroxyl (—OH) groups.

This synthesized ZnO-PBA-Curcumin could maintain a strong drug-nanoparticle interaction under physiological conditions and exhibited a pH-responsive drug release behavior. Complexations of curcumin with divalent zinc ions and zinc oxide nanoparticles have been employed here to load curcumin onto the ZnO-PBA NPs surface and to increase its stability [39]. However, curcumin was released from the nanohybrid when the breakdown of the complex occurred. At low pH, the Zn^2+^-Curcumin complex is less stable, as curcumin is predominantly present in its unionized form (poor ligand) in addition to the partial dissolution of ZnO NPs. This pH-responsive drug delivery system would not only diminish the amount of drug loss to the blood circulation, but it would also help in drug release following the endocytosis process, thereby improving the therapeutic efficacy.

Fluorescence microscopy and flow cytometry showed that ZnO-PBA had a higher level of cellular uptake in cancer cells compared to ZnO NPs. Cancer cells express higher concentrations of sialic acid on their membranes compared to normal cells [40]. The overexpressed sialic acid accelerates cellular proliferation and metastasis. ZnO-PBA has a higher accumulation in cancer cells because of the interaction of PBA with sialic acid via the formation of boronate esters [41].

The *in vitro* cytotoxicity experiments and morphological analyses confirmed that ZnO-PBA-Curcumin exhibited higher tumor cell growth inhibition over free curcumin and ZnO NPs at all the equivalent concentrations tested in MCF-7 breast cancer cells. To investigate the underlying mechanism behind the enhanced cytotoxic potential of ZnO-PBA-Curcumin, the intracellular ROS levels and MMP were checked. At the basal level, ROS are helpful for assisting in cell growth and differentiation. However, accumulation of excessive ROS (oxidative stress) causes the impairment of several vital cellular functions and leads to cell death. Oxidative stress downregulates MMP and causes an imbalance of anti- and pro-apoptotic proteins, leading to the activation of extrinsic apoptosis [42]. In the present study, ZnO-PBA-Curcumin causes oxidative stress as well as mitochondrial dysfunction, leading to cellular apoptosis. However, ZnO-PBA-Curcumin exposure showed the combined effects of both ZnO and curcumin towards oxidative stress, mitochondrial dysfunction and cytotoxicity, and therefore, it can be considered as a potential candidate for cancer chemotherapy.

Previous literature also suggests that 3-mercaptopropionic acid (MPA) can be used as a linker between ZnO NPs and curcumin [6]. Unfortunately, the authors of this study did not mention anything about the release of the covalently bound drug molecule (curcumin) in a condition mimicking cancer cells (low pH). There was also no active targeting of the nanohybrid to the cancer cells. In our present study, curcumin was loaded through the formation of a chelate ring with ZnO, which showed pH-dependent release properties via the breaking of the chelate complex. PBA was conjugated through a covalent bond with the ZnO NPs and acts as a tumor-targeting molecule.

An *in vivo* mouse model was developed and used to validate the anticancer efficacy of the nanohybrid. Here, also, ZnO-PBA-Curcumin treatment was found to be more effective in showing significant antitumor activity compared to equivalent amounts of ZnO NPs and free curcumin. ZnO NPs not only made curcumin more stable but also directed it selectively into tumor tissues, thereby synergistically enhancing its antitumor efficacy.

Malignancy induces high proliferation rates and the emergence of multinucleated tumor cells within the tissue. Many multinucleated tumor cells were visible in the histological section of the untreated tumor tissue. These multiple nuclei were seen to reduce in number with ZnO-PBA, curcumin, and ZnO-PBA-Curcumin treatment, indicating the success of the treatments. The ZnO-PBA-Curcumin treatment was seen to be the most successful in this regard, with the maximum reduction in the number of multinucleated tumor cells among the three treated groups. Additionally, normal muscle tissue architecture was seen to be completely lost in the untreated tumor tissue. However, the muscle tissue architecture began to return to normal with curcumin and ZnO-PBA-Curcumin treatment, with ZnO-PBA-Curcumin treatment again being the most successful, as can be seen in the Fig. 5D.

The biocompatibility of the drug delivery system is another important issue for cancer therapy study. For an *in vivo* safety assessment, ZnO and ZnO-PBA-Curcumin were injected via tail vein to both tumor-bearing mice and control mice. Biochemical as well as histological analyses were performed. It is encouraging that the *in vivo* safety study did not show any signs of hepatic and renal toxicity after intravenous injection of the nanoparticles. The histological study was done to detect any toxic effects of the nanoparticle compounds *in vivo*. All of the liver and kidney samples for histology analysis were sourced from mice bearing no tumor that were treated with nanoparticles. In both the liver as well as kidney sections, no significant changes were recorded, indicating minimal to no toxicity to these vital body organs. The liver sinusoids and the central vein in the liver tissue of the treated mice were seen to be just as intact as in the untreated mice. Similarly, the glomeruli and renal cords were seen to be intact in the nanoparticle-treated mice, similar to the untreated mice.

The reduction in splenomegaly in the tumor-bearing animals may be due to the anti-inflammatory properties of curcumin [43]. In the present study, tumor induction with EAC cells in mice caused spleen enlargement. This seemed to be a potential side effect of tumor induction. However, treatment with ZnO nanoparticles, free curcumin and the curcumin-loaded ZnO nanoparticles did not show any additional splenotoxicity compared to the untreated group, as indicated by no further increase in spleen size. Moreover, the nanohybrid was able to show anticancer properties to the extent that the spleen was restored to normal size, indicating a restoration of normal physiology.

Overall, the obtained data showed that at the used dose, free curcumin and the NPs in the experimental protocol are physiologically suitable and have no toxic effects on the other vital organs of a living system. Based on its enhanced antitumor efficiency and higher biocompatibility, ZnO-PBA-Curcumin could be considered as a novel therapeutic approach for breast cancer treatment.

## Conclusions

In the present work, ZnO-PBA-Curcumin was formulated as a drug delivery system for cancer cells. Due to PBA conjugation with nanoparticles, the absorption of the drug molecule is increased in tumor tissue through the interaction with sialic acid (which is overexpressed in the cancer cells), i.e., it exhibited tumor-targeting ability. It also exhibited high drug loading and release profiles, displayed clear advantages and significantly enhanced *in vitro* and *in vivo* antitumor efficacy. ZnO dissociates at low pH values, i.e., curcumin is released more in tumor cells than in normal cells. Overall, this study offers an improved, targeted tumor therapy strategy for breast cancer treatment without systemic toxicity, which might also be used in an advanced way in the future.

## Conflict of interest

*The authors have declared no conflict of interest.*
