# Supplementary material for: pH-responsive and targeted delivery of curcumin via phenylboronic acid-functionalized ZnO nanoparticles for breast cancer therapy
Source: J Adv Res. 2019 Mar 1;18:161–72. doi: 10.1016/j.jare.2019.02.036 (PMC6479012; doi:10.1016/j.jare.2019.02.036)
Supplement: Supplementary data 1 [file mmc1.doc]

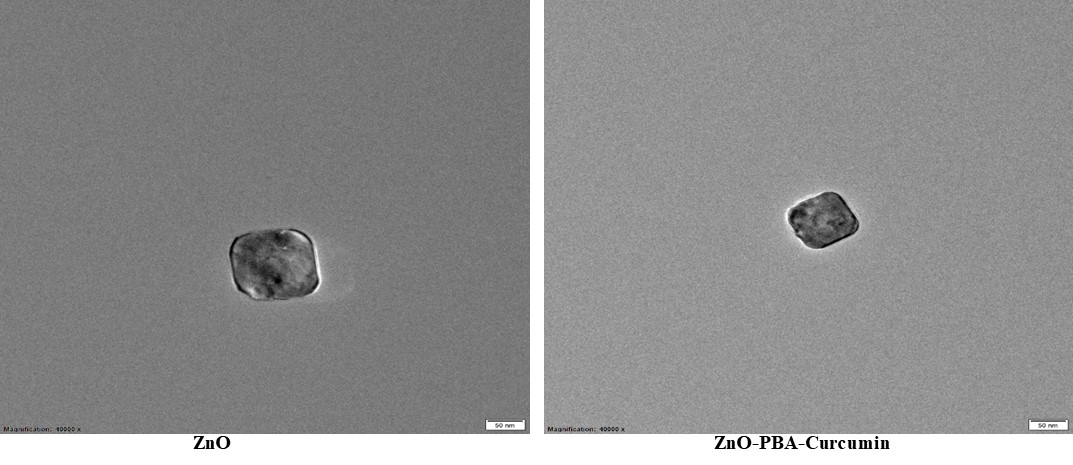
 **Fig.S1: TEM Analysis**


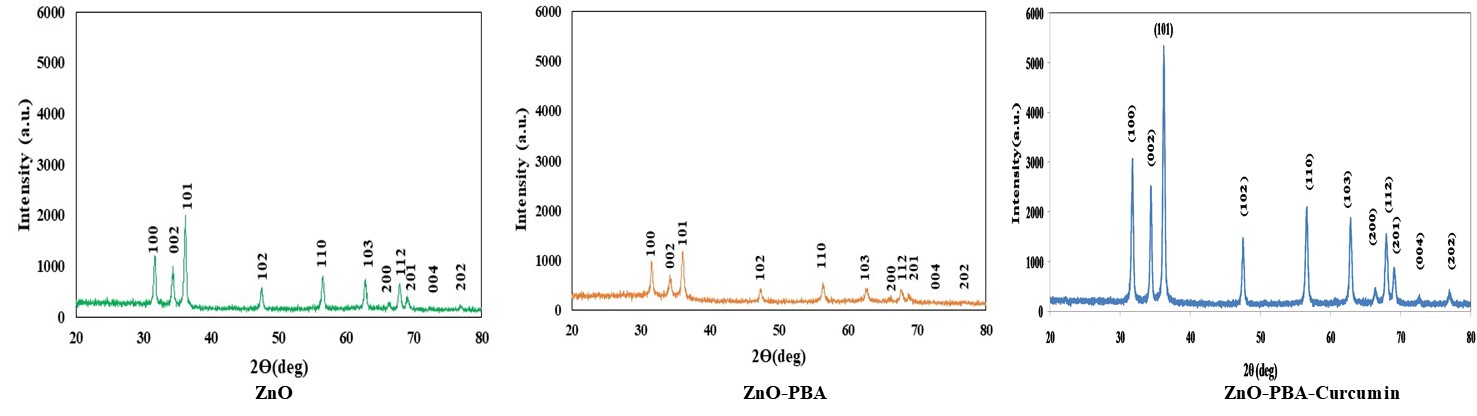
 **Fig. S2: XRD Analysis**

**
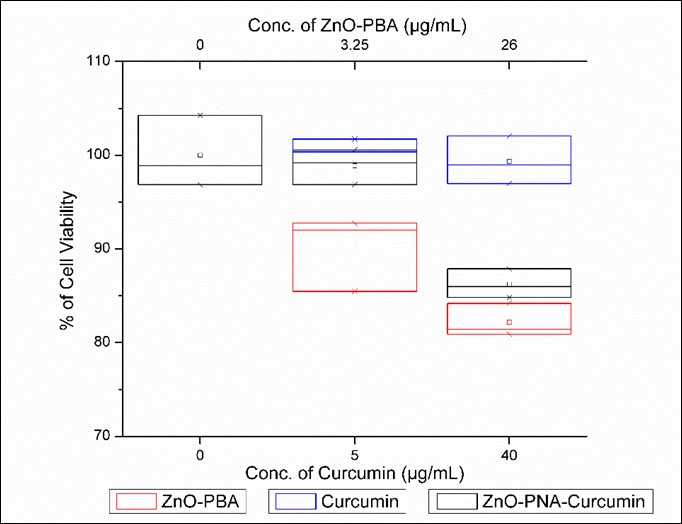
**

**Fig.S3: Cell viability assay of normal cells**

Table S1: DLS of ZnO, ZnO-PBA and ZnO-PBA-Curcumin

| **DLS** | **ZnO** | **ZNO-PBA** | **ZnO-PBA-Curcumin** |
| --- | --- | --- | --- |
| **Mean** | **166.3** | **284.9667** | **413.6333333** |
| **SD** | **7.901899** | **8.300803** | **9.584536156** |

**Table S2: Zeta Potential of ZnO, ZnO-PBA and ZnO-PBA-Curcumin**

| **Zeta-potential** | **ZnO** | **ZnO-PBA** | **ZnO-PBA-Curcumin** |
| --- | --- | --- | --- |
| **Mean** | **17.9** | **-4.76667** | **-16.4** |
| **SD** | **0.2** | **0.305505** | **0.3** |

Table S3: Bio-distribution of curcumin in tumor bearing animals

| **Bio-distribution** | **Curcumin** | **ZnO-Curcumin** | **ZnO-PBA-Curcumin** |
| --- | --- | --- | --- |
| **Mean** | **4.117666667** | **13.45** | **24.63** |
| **SD**  ***P*=0.00000896532** | **1.519933003** | **1.891533769** | **0.908129947** |

**Table S4: Tumor growth volume**

| **Tumor growth volume** | **Untreated** | **ZnO-PBA** | **Curcumin** | **ZnO-PBA-Curcumin** |
| --- | --- | --- | --- | --- |
| **0** | **2.62667** | **2.47333** | **2.40333** | **2.64667** |
| **5** | **3.70667** | **3.32** | **3.28667** | **2.83667** |
| **10** | **6.74667** | **4.55667** | **3.64** | **2.28333** |
| **14** | **9.22167** | **5.56283** | **3.49983** | **2.21833** |
|  |  |  |  |  |
| **SD** | **0.28537** | **0.3421** | **0.24502** | **0.17786** |
|  | **0.14572** | **0.54809** | **0.29023** | **0.21939** |
|  | **0.48398** | **0.26502** | **0.37323** | **0.27025** |
|  | **0.95336** | **1.1888** | **0.15505** | **0.51991** |

**Table S5: Tumor mass**

| **Tumor mass** | **Tumor control** | **ZnO-PBA** | **Curcumin** | **ZnO-PBA-Curcumin** |
| --- | --- | --- | --- | --- |
| **Mean** | **8.6972** | **5.990233** | **4.594167** | **2.3876** |
| **SD**  ***P*=0.000054104** | **0.285876** | **0.75575** | **0.264464** | **0.229774** |

Table S6: ALP

| **ALP** | **Control** | **Tumor untreated** | **ZnO-PBA treated tumor** | **Curcumin treated tumor** | **ZnO-PBA-Curcumin treated tumor** | **ZnO-PBA treated** | **Curcumin treated** | **ZnO-PBA-Curcumin treated** |
| --- | --- | --- | --- | --- | --- | --- | --- | --- |
| **Mean** | **63.48342** | **126.4276633** | **122.6554** | **77.38333333** | **116.13571** | **65.61403333** | **61.58046667** | **63.36906667** |
| **SD**  **P=1.45534062892928E-17** | **0.815692** | **0.589102624** | **2.245489018** | **3.499602821** | **3.521290311** | **0.900178318** | **0.965041275** | **2.835317127** |

**Table S7: ALT**

| **ALT** | **Control** | **Tumor untreated** | **ZnO-PBA treated tumor** | **Curcumin treated tumor** | **ZnO-PBA-Curcumin treated tumor** | **ZnO-PBA treated** | **Curcumin treated** | **ZnO-PBA-Curcumin treated** |
| --- | --- | --- | --- | --- | --- | --- | --- | --- |
| **Mean** | **25.89187** | **41.70453333** | **41.15996667** | **35.12986667** | **38.93273333** | **26.5633** | **25.52963333** | **25.5929** |
| **SD**  ***P*=0.00000000000001532** | **0.928198** | **0.48489328** | **0.860603052** | **1.605032502** | **0.248571304** | **0.544397291** | **0.845544797** | **0.866138557** |

**Table S8: Creatinine**

| **Creatinine** | **Control** | **Tumor untreated** | **ZnO-PBA treated tumor** | **Curcumin treated tumor** | **ZnO-PBA-Curcumin treated tumor** | **ZnO-PBA treated** | **Curcumin treated** | **ZnO-PBA-Curcumin treated** |
| --- | --- | --- | --- | --- | --- | --- | --- | --- |
| **Mean** | **0.395733333** | **0.842566667** | **0.732433333** | **0.488966667** | **0.691166667** | **0.456633333** | **0.4037** | **0.474733333** |
| **SD**  ***P*=0.0000000000348171** | **0.015923044** | **0.065921645** | **0.007905273** | **0.012706429** | **0.040114877** | **0.04520236** | **0.006502307** | **0.017003039** |

Table S9: BUN

| **Bun** | **Control** | **Tumor untreated** | **ZnO-PBA treated tumor** | **Curcumin treated tumor** | **ZnO-PBA-Curcumin treated tumor** | **ZnO-PBA treated** | **Curcumin treated** | **ZnO-PBA-Curcumin treated** |
| --- | --- | --- | --- | --- | --- | --- | --- | --- |
| **Mean** | **11.70393** | **43.71899** | **39.3238** | **29.18397** | **35.22603** | **12.1473** | **11.41737** | **11.93643** |
| **SD**  ***P*=0.001821126484584** | **0.35097** | **0.75201** | **0.4659** | **0.91002** | **1.1843** | **0.12831** | **0.23115** | **0.10178** |
